# COVID-19 Response Resource Engagement and User Characteristics of the Wichealth Web-Based Nutrition Education System: Comparative Cross-sectional Study

**DOI:** 10.2196/38667

**Published:** 2023-03-02

**Authors:** John J Brusk, Robert J Bensley

**Affiliations:** 1 School of Interdisciplinary Health Programs Western Michigan University Kalamazoo, MI United States

**Keywords:** COVID-19, user engagement, infodemic, Women, Infants, and Children, WIC, educational resource, health care, digital health, nutrition, web-based education, web-based nutrition, pediatric, parenting, dashboard

## Abstract

**Background:**

In response to the COVID-19 pandemic, Wichealth launched 4 information resources on the site’s user landing dashboard page. These resources were used consistently during the period in which they were available (April 1, 2020, through October 31, 2021); however, only 9% (n=50,888) of Wichealth users eligible for inclusion in the study accessed at least one resource. User engagement with emergency response resources within the context of a web-based health educational tool has not been well investigated due to a paucity of opportunities and a lack of the ability to evaluate relevant users at scale.

**Objective:**

This investigation was carried out to understand if user characteristics and behaviors measured by the Wichealth web-based education system are associated with a participant's motivation, or lack thereof, to engage with the added COVID-19 resources.

**Methods:**

Sociodemographic characteristics were gathered from Wichealth users with at least one lesson completed and a complete user profile to identify which factors increase the likelihood of user access of any of the Wichealth COVID-19 response resources during the 19-month period between April 1, 2020, and October 31, 2021. A logistic regression analysis was conducted to determine the relative importance of all factors on the likelihood of a user accessing the COVID-19 resources.

**Results:**

A total of 50,888 unique Wichealth users included in the study accessed the COVID-19 response resources 66,849 times during the time period. During the same period, 510,939 unique Wichealth users completed at least one lesson about how to engage in healthy behaviors with respect to parent-child feeding but did not access any COVID-19 resources. Therefore, only 9% of Wichealth users who completed a lesson during the time when COVID-19 response resources were available accessed any of the information in those resources. Users of the Spanish language Wichealth version, older users, those less educated, and users with prior Wichealth lesson engagement demonstrated the greatest likelihood of COVID-19 resource use.

**Conclusions:**

This investigation presents findings that demonstrate significant differences between Wichealth users that opted to access COVID-19–specific resources and those who chose not to during their web-based educational session. Reaching users of a web-based educational system with supplemental information may require multiple strategies to increase coverage and ensure the widest possible distribution.

## Introduction

Wichealth is a web-based health behavior change system, with versions in both English and Spanish, for families who participate in the Special Supplemental Nutrition Program for Women, Infants, and Children (WIC) [1]. Currently, Wichealth serves 32 states and 15 Indian Tribal Organizations, from where over 500,000 unique individuals complete over 1 million lessons each year. Nearly 85% of Wichealth users indicated that the web-based system is their preferred method of receiving nutrition and parent-child feeding education. Further, over 95% of clients who log onto Wichealth fully complete at least one lesson during their visit. This is driven by the need for WIC clients to complete the secondary education contact requirement associated with food benefits distribution. In comparison, nonrequired resources available on the site, such as the Wichealth Health eKitchen recipe and video library, were accessed by over 12% of those completing lessons during the period under study. Previous studies have reported the positive impact and participant engagement associated with Wichealth usage [2,3].

In response to the COVID-19 pandemic, Wichealth launched 4 information resources on the site’s user landing dashboard page. The expectation was that Wichealth users would want access to information about how COVID-19 would impact their WIC benefits or other parent-child feeding concerns. Although these resources were used consistently during the period in which they were available (April 1, 2020, through October 31, 2021), only about 9% of Wichealth users accessed at least one resource. This investigation was carried out to understand factors associated with a participant’s motivation, or lack thereof, to engage with these resources. User engagement with emergency response resources within the context of a web-based health educational tool has not been well investigated due to a paucity of opportunities and a lack of the ability to evaluate relevant users at scale.

Prior research investigating the reasons for individual engagement with health-related information suggests many personal characteristics are associated with user interaction with web-based systems, such as culture, race, age, and education [4]. An investigation of the general US population found that significantly higher levels of web-based health information engagement is associated with younger ages and higher levels of education [5]. These findings are supported by the results of another study where participants were included via Facebook recruitment [6]. In this study, the investigators found that users in the youngest and most educated groups were more likely to report they would use web-based sources for COVID-19 information. In another study comprised of WIC clients, researchers found that college graduates were more likely to engage with authoritative sources, whereas others had an increased preference for participating in community forums or other social media platforms [7]. Song and colleagues previously reported similar findings with respect to low income and undereducated, expectant mothers’ engagement with health information [8]. They found that study participants preferred to seek out information from trusted individuals around them, most notably their family and the father of the baby. Further, these women infrequently used web-based sources for health information, as getting information from family further contributed to their perceived increased level of support and reduced uncertainty about having a baby. However, nonpregnant users with larger families for whom they provide care, tend to be avid web-based health information consumers [9]. In more recent articles, sociodemographic characteristics strongly associated with digital health information seeking included socioeconomic status, age, ethnicity, and college-level education [10,11]. Further, another recent analysis found that self-efficacy had positive effects on the extent of learner engagement with educational resources [12].

## Methods

### Procedure

Characteristics of Wichealth users engaging with the emergency COVID-19 response resources were compared with Wichealth users who did not record any COVID-19 uses during the period between April 1, 2020, and October 31, 2021, when the resources were available. Data were gathered from users with a completed Wichealth demographic profile and at least one exit survey following completion of a web-based lesson. Wichealth profile characteristics include user race, ethnicity, pregnancy status, educational level, marital status, and age group. Wichealth exit survey metrics that describe how the user interacted with the lesson they most recently completed were also included. These consist of the language version of the lesson most recently completed, the category of lesson completed, the type of device on which the lesson was completed, whether the user actively engaged with the lesson completed, user feedback about their preference using Wichealth for learning about the topic, user feedback about self-efficacy with respect to applying what they learned, and user feedback about whether they would recommend the lesson to others. Active user engagement was determined based on whether a user, while completing the lesson, performed at least one interaction event, including “liking” a resource, “favoriting” (bookmarking) a resource, or sharing a resource with a friend. User self-efficacy was based on whether the user believed they could make healthy changes using what they learned from the most recently completed lesson. Users with missing profile data or without at least one lesson completion, including exit survey, were not included in this study. A logistic regression analysis was conducted to determine the relative importance of all factors on the likelihood of a user accessing the COVID-19 resources. The python open-source statistical packages Scikit-learn version 0.19.2 [13] and SciPy version 1.1.0 [14] were used to determine the best fit model coefficients and standard errors to calculate odds ratios (ORs) and confidence intervals describing the extent to which each factor was associated with COVID-19 resource access, adjusted for other factors considered by the model. Binary response variables were coded as single model features, and multiclass variables were each one-hot encoded into binary model features for each class.

### Ethics Approval

Human subject review board approval (Western Michigan University Human Subjects Institutional Review Board, WMU HSIRB 13-09-06) for use of Wichealth system’s collected data to better understand reach, impact, and opportunities for innovation has been in place for nearly two decades. Users who complete profiles when accessing Wichealth do so agreeing to allow use of the data for system improvement. No individually identifiable information was retrieved about users during this analysis. All data are stored in a database with multifactor authentication, which is only accessible by the study investigators. No compensation was received by the primary investigator for this work. The data analyst received minimal compensation to cover cost of compute, tool setup, and data hosting.

## Results

There was a total of 770,290 users who accessed Wichealth during the time period between April 1, 2020, and October 31, 2021. In total, 561,827 (72.9%) had completed profile data and at least one lesson with associated exit survey completed; 50,888 (9.1%) of these Wichealth users accessed the COVID-19 response resources at least once for a total of 66,849 times during the period they were available (Table 1), and 510,939 (90.9%) recorded no COVID-19 response resource use.

The distribution of several Wichealth user characteristics were markedly different when comparing those who accessed COVID-19 resources to those who did not (Table 2). Results of the best fit logistic regression model were 79% accurate in predicting COVID-19 resource use in the test set and explained 66% of the test sample variance. The model identified significant differences between several user factors, controlling for the effect of other user characteristics in the model. Spanish language COVID-19 resource use was nearly double that of Wichealth English version users (n=4564, 9% vs n=23,429, 4.6%). This difference was strongly significant controlling for other user factors (OR 1.37, 95% CI 1.29-1.45).

Latino ethnicity only had a marginal effect on COVID-19 resource use (OR 1.06, 95% CI 1.01-1.12). User race demonstrated more importance with respect to COVID-19 resource use with Asians (OR 1.11, 95% CI 1.08-1.14), and Native Hawaiian or Pacific Islanders (OR 1.16, 95% CI 1.11-1.21) were moderately associated with a higher likelihood of use. The White users (OR 0.79, 95% CI 0.77-0.81) and Black or African Americans (OR 0.73, 95% CI 0.73-0.78), however, were much less likely to access the supplemental resources.

Users who were pregnant were more likely to access COVID-19 resources (OR 1.15, 95% CI 1.12-1.17). Marital status (OR 1.09, 95% CI 1.05-1.12) was also marginally important, as married users were slightly more likely to use a COVID-19 resource when controlling for other factors.

Educational status was also related to user COVID-19 resource access, with lower levels of education tending to be more likely to record usage. This includes users who did not finish high school (OR 1.03, 95% CI 1.01-1.04) and users for whom high school was the highest level of education (OR 1.04, 95% CI 1.02-1.07).

Older Wichealth age groups were much more likely to access the COVID-19 resources with users aged 40-49 years (OR 1.06, 95% CI 1.02-1.10) and uses older than 50 years (OR 1.53, 95% CI 1.48-1.58) most likely to record access.

Characteristics of how users interacted with their most recently completed lesson also demonstrated some importance with respect to access to the COVID-19 resources. The most important factor associated with a user’s likelihood of COVID-19 resource access was their prior engagement with the Wichealth lesson they most recently completed by liking, bookmarking, or sharing the lesson resource (OR 1.31, 95% CI 1.28-1.35). Users who indicated they would recommend their most recently completed lesson to others (OR 1.09, 95% CI 1.03-1.14) and users who indicated they believed they could make changes using what they learned from the lesson (OR 1.10, 95% CI 1.06-1.14), a measure of self-efficacy, also were more likely to use the resources. However, users who indicated that Wichealth was their preferred resource for nutrition education were less likely to use the COVID-19 resources (OR 0.87, 95% CI 0.81-0.94). The category of the lesson most recently completed was also a factor, with users having recently completed lessons in the “Pregnancy and Baby’s First 6 Months” (OR 1.16, 95% CI 1.13-1.18) and the “Mothers in Motion” (OR 1.33, 95% CI 1.27-1.38) categories being more likely to access the resources.

**Table 1:** Wichealth COVID-19 resource uses by resource title (April 2020 to October 2021).

Wichealth COVID-19 resource title	Total uses, n (%)
Shopping tips in case of COVID-19 shortages	18,185 (27.2)
Meal planning during COVID-19	17,321 (25.9)
Sources you can trust for COVID-19 information	18,320 (27.4)
COVID-19 guidance: feeding your baby	13,023 (19.5)
Any COVID-19 resource use	66,849 (100)

**Table 2:** Distribution and odds of Wichealth user factors by study group (April 2020 to October 2021).

User characteristic		Wichealth users with COVID-19 resource uses, n (%)	Wichealth users with no COVID-19 resource uses, n (%)	Logistic regression model weight (Coefficient)	Odds ratio	SE	95% CI lower bound	95% CI upper bound
**Race**								
	White	29,546 (58.1)	309,075 (60.5)	–0.24	0.79	0.01	0.77	0.81
	Black or African American	7629 (15)	87,100 (17)	–0.28	0.76	0.01	0.73	0.78
	Multiracial	5257 (10.3)	50,256 (9.8)	–0.01	0.98	0.02	0.95	1.01
	Asian	5035 (9.9)	38,406 (7.5)	0.11	1.11	0.02	1.08	1.14
	Native American or Alaskan	2000 (3.9)	15,965 (3.1)	0.01	1.01	0.02	0.97	1.05
	Native Hawaiian or Pacific Islander	1421 (2.8)	10,137 (2)	0.15	1.16	0.03	1.11	1.21
**Latino ethnicity**								
	No	33,745 (66.3)	354,579 (69.4)	Ref^a^	Ref	Ref	Ref	Ref
	Yes	17,143 (33.7)	156,360 (30.6)	0.03	1.06	0.03	1.01	1.12
**Pregnant**								
	No	43,764 (86)	448,657 (87.8)	Ref	Ref	Ref	Ref	Ref
	Yes	7124 (14)	62,282 (12.2)	0.14	1.15	0.01	1.12	1.17
**Education**								
	High school degree or GED^b^	19,528 (38.4)	184,913 (36.2)	0.03	1.03	0.01	1.01	1.04
	Some college	12,155 (23.9)	138,870 (27.2)	–0.16	0.85	0.01	0.83	0.87
	4-year college or university degree	5751 (11.3)	57,729 (11.3)	–0.15	0.86	0.01	0.84	0.89
	Did not finish high school	6198 (12.2)	51,973 (10.2)	0.04	1.04	0.01	1.02	1.07
	Community college degree	3685 (7.2)	41,292 (8.1)	–0.17	0.84	0.02	0.81	0.87
	Trade skills training	2159 (4.2)	23,616 (4.6)	–0.19	0.82	0.02	0.78	0.87
	Advanced college degree	1412 (2.8)	12,546 (2.5)	–0.02	0.98	0.02	0.93	1.03
**Marital status**								
	Single or never married	25,666 (50.4)	277,944 (54.4)	–0.15	0.86	0.02	0.83	0.89
	Married	21,483 (42.2)	196,010 (38.4)	0.08	1.09	0.02	1.05	1.12
	Divorced	3484 (6.8)	34,561 (6.8)	0.03	1.03	0.02	0.99	1.07
	Widowed	255 (0.5)	2424 (0.5)	0.01	1.01	0.06	0.89	1.13
**Age group**								
	≤18	223 (0.4)	3534 (0.7)	–0.70	0.50	0.06	0.38	0.61
	19-29	19,721 (38.8)	228,780 (44.8)	–0.31	0.74	0.02	0.70	0.77
	30-39	23,360 (45.9)	220,900 (43.2)	–0.11	0.90	0.02	0.86	0.93
	40-49	6125 (12)	48,556 (9.5)	0.06	1.06	0.02	1.02	1.10
	≥50	1459 (2.9)	9169 (1.8)	0.43	1.53	0.03	1.48	1.58
**Wichealth language version**								
	English	46,324 (91)	487,510 (95.4)	Ref	Ref	Ref	Ref	Ref
	Spanish	4564 (9)	23,429 (4.6)	0.14	1.37	0.04	1.29	1.45
**Prefer Wichealth for nutrition education**								
	No	6644 (13.1)	59,815 (11.7)	Ref	Ref	Ref	Ref	Ref
	Yes	44,244 (86.9)	451,124 (88.3)	–0.06	0.87	0.03	0.81	0.94
**Recommend Wichealth to others**								
	No	1,270 (2.5)	14,172 (2.8)	Ref	Ref	Ref	Ref	Ref
	Yes	49,618 (97.5)	496,767 (97.2)	0.04	1.09	0.03	1.03	1.14
**Believe can make healthy change**								
	No	3042 (6.0)	32,954 (6.4)	Ref	Ref	Ref	Ref	Ref
	Yes	47,846 (94.0)	477,985 (93.6)	0.04	1.10	0.02	1.06	1.14
**Wichealth engagement recorded**								
	No	45,138 (88.7)	469,101 (91.8)	Ref	Ref	Ref	Ref	Ref
	Yes	5750 (11.3)	41,838 (8.2)	0.27	1.31	0.02	1.28	1.35
**Device used to use Wichealth**								
	Mobile	40,823 (80.2)	396,049 (77.5)	0.01	1.03	0.01	1.00	1.06
	Desktop	9221 (18.1)	108,699 (21.3)	–0.08	0.84	0.02	0.81	0.87
	Tablet	844 (1.7)	6191 (1.2)	0.08	1.21	0.03	1.16	1.27
**Wichealth lesson category**								
	Keeping your family healthy	11,289 (22.2)	112,118 (21.9)	0.00	1.00	0.01	0.97	1.03
	Choosing healthy foods	8999 (17.7)	88,816 (17.4)	0.03	1.07	0.01	1.04	1.10
	Feeding your 6- to 24-month-old baby	6367 (12.5)	77,601 (15.2)	–0.06	0.86	0.01	0.83	0.89
	Pregnancy and baby’s first 6 months	8632 (17)	77,051 (15.1)	0.06	1.16	0.01	1.13	1.18
	Feeding your 2- to 5-year-old baby	5284 (10.4)	59,209 (11.6)	–0.06	0.86	0.02	0.83	0.90
	Planning simple meals and snacks	5312 (10.4)	47,807 (9.4)	0.04	1.10	0.02	1.07	1.13
	Welcome to WIC^c^	1340 (2.6)	17,179 (3.4)	–0.14	0.73	0.03	0.67	0.79
	Mothers in motion	1814 (3.6)	14,012 (2.7)	0.12	1.33	0.03	1.27	1.38
	New and expecting parents	988 (1.9)	8159 (1.6)	0.06	1.14	0.03	1.08	1.20
	Understanding developmental milestones	486 (1)	5802 (1.1)	–0.03	0.93	0.04	0.84	1.01
	Healthy families	377 (0.7)	3185 (0.6)	0.05	1.13	0.05	1.03	1.22

^a^Ref: reference.

^b^GED: General Educational Development.

^c^WIC: Women, Infants, and Children.

## Discussion

### Principal Findings

This investigation presents findings that demonstrate significant differences between Wichealth users who opted to access COVID-19–specific resources and those users who did not. Spanish language users were more likely to engage with COVID-19 response resources. Spanish language Wichealth users made up 9% of the COVID-19 response resource views, more than twice the overall rate of Wichealth English version usage. These findings support prior research that demonstrated higher levels of engagement for Spanish-speaking users [3,11]. Other user characteristics with a greater likelihood of COVID-19 resource access included older age, lower levels of education, and affirmative married or partner status.

User Wichealth behavioral characteristics were also presented with respect to differences in rates of COVID-19 response resource use. Wichealth users who were more likely to engage with educational resources during their more recently completed Wichealth lesson were more likely to engage with the COVID-19 resources. Previous findings indicate web-based educational resource engagement is positively associated with higher levels of education and marital status [12]. However, the results reported in this study suggest that higher education levels had markedly lower levels of COVID-19 information seeking. Additional Wichealth user behavioral metrics compared—such as recommendation of the lesson to others and belief in their own ability to make healthy changes, given what they learned—each demonstrated a positive relationship with the likelihood of COVID-19 resource access.

The COVID-19 emergency response resources were launched to serve as a means for addressing Wichealth users’ pandemic uncertainty, enabling them to move forward with learning objectives despite emerging COVID-19 concerns. Use of these resources was relatively low compared to other supplemental Wichealth resources, such as the Health eKitchen recipe and video library, which draws usage from over 12% of Wichealth log-ins. Further, by comparison, these resources would not be among the top 50 used during the time period compared to individual resources available throughout each Wichealth lesson. These results support that, despite the availability of relevant information from an already trusted, credible source, the vast majority (n=510,939, 91%) of users were not motivated to access them.

### Limitations

The primary limitation of this study is that comparison group membership is based on self-selection; however, as the goal was to understand differences among those selecting to use COVID-19 resources compared to those who did not, this is of minor impact. Further, only users with lesson completions were included in the study. This is because Wichealth key performance indicators are only fully assessed at the conclusion of a lesson. Additionally, due to the relatively few COVID-19 resources used, no attempt was made to determine how the particular COVID-19 topic addressed by each resource was impacted by the factors associated with a user’s likelihood of accessing any of the COVID-19 resources.

### Conclusions

Results of the analysis of Wichealth key performance indicators for lessons completed among users who accessed COVID-19 response resources compared to those who did not access them demonstrated many key differences. As expected, users who recorded engagement with their most recently completed Wichealth lesson were significantly more likely to record usage of the COVID-19 response resources. This suggests that groups with a prior system track record of low engagement can be expected to engage less with any new resources or supplemental information made available. As demonstrated previously, Spanish Wichealth version users were much more likely to access the COVID-19 resources, and likewise, they were twice as likely to engage with the lessons they complete. These 2 factors, along with users’ older age and marital status, were the most important drivers of the COVID-19 response resource use. When users indicated they would not recommend the lesson they most recently completed to others, it may reflect a higher level of dissatisfaction with the lesson, negatively impacting the likelihood that the user accesses additional or supplemental resources. Moreover, individuals who accessed the COVID-19 response resources were slightly more likely to believe they could make changes using what they learned from their most recently completed lesson, controlling for other factors.

Reaching users of a web-based educational system with supplemental information may require multiple strategies to increase coverage and ensure the widest possible distribution. Making supplemental resources available may have resonated with users more likely to engage with web-based information but may have left behind those using Wichealth only to complete educational requirements with little or no engagement. These users may benefit from more strategically placed and integrated supplemental information within the context of the Wichealth lesson they are required to complete.

## Abbreviations

OR: odds ratio
WIC: Women, Infants, and Children
